# Phenotype and Tissue Residency of Lymphocytes in the Murine Oral Mucosa

**DOI:** 10.3389/fimmu.2017.00250

**Published:** 2017-03-08

**Authors:** Joo-Young Park, Hyunsoo Chung, Youngnim Choi, Jung-Hyun Park

**Affiliations:** ^1^Experimental Immunology Branch, National Cancer Institute, NIH, Bethesda, MD, USA; ^2^Department of Oral Microbiology and Immunology, School of Dentistry and Dental Research Institute, Seoul National University, Seoul, South Korea

**Keywords:** cellular immunity, maxillofacial, collagenases, flow cytometry, monocytes, cell isolation

## Abstract

The oral mucosa is a critical barrier tissue that harbors a series of distinct immune cell subsets. Immune surveillance in the oral mucosa is important for both local and systemic immunity because the oral cavity is a heavily utilized route of pathogen entry and also serves as site of pathogen propagation. Nonetheless, composition and phenotype of the lymphocyte pool in the oral mucosa have remained poorly characterized. Utilizing a newly established protocol for mucosal immune cell isolation, here, we report that the oral mucosa features a unique cellular composition of immune cells, which differed not only from secondary lymphoid organs but also from mucosal tissues in the gut and lung. We observed profound accumulation of CD11b^+^Ly6C^lo^ monocytes in the oral mucosa that were maintained independently of T- and B-lymphocytes. Unlike the gut mucosa, the oral mucosa neither contained CD8αα T cells nor was it enriched for CD103^+^CD69^+^ tissue-resident memory CD8 T cells. In fact, a major fraction of T cells circulated and trafficked through the mucosa as revealed by treatment with the S1P1 receptor antagonist, FTY720, a potent inhibitor of lymphocyte migration. Collectively, these results provide a comprehensive picture of immune cells in the oral mucosa as an active site of lymphocyte recruitment and surveillance.

## Introduction

The oral cavity is one of the most frequently exposed sites to foreign antigens as it constantly encounters food-borne, water-borne, and air-borne antigens and other environmental insults (1–3). As such, it is astonishing that the oral cavity is normally absent of inflammation, and that fungal and other microbiological infections rarely happen under steady-state conditions (4). Multiple pathways have been attributed to achieve this feature, including production of antimicrobial peptides, expression of proteolytic enzymes, and also antibody secretion (5–8). As another major mechanism, it is understood that the oral mucosa, which lines the oral cavity, serves as a highly effective barrier tissue to filter and fight foreign pathogens and that it also suppresses overt and excessive immune reactions to maintain an effective but quiescent immune system (4, 9).

The oral mucosa is composed of two structural layers: the outer epithelium and the underlying lamina propria (LP) (10). While the epithelium primarily serves as a physical and chemical barrier, immune cells scattered through the epithelium and LP constitute an immunological barrier that scavenges invading microbes/antigens and initiates protective immune reactions (11, 12). Immune surveillance in the oral mucosa is orchestrated by interplay of tissue-resident and migratory cells that triggers humoral and cellular immune responses by lymphocytes and other hematopoietic origin cells. Conventionally, resident cells in the mucosa are understood as cells of stromal origin, such as gingival keratinocytes, fibroblasts, and also periodontal ligament cells. Migratory cells, on the other hand, are primarily of lymphoid origin and also include circulating granulocytes, such as neutrophils (13). However, with the identification of tissue-resident memory T cells (14–16), and the discovery of migratory CD103^+^ dendritic cells in non-lymphoid tissues (17), the partition into migratory and resident cells has become less clear.

While the characteristics of antigen presenting cells (APCs) in the oral mucosa, such as Langerhans cells, dendritic cells, and macrophages, have been studied to some extent (18–22), our knowledge on the cellular composition of immune cells and tissue residency of lymphoid cells in the oral mucosa remains limited. A major obstacle to address these issues has been the failure to efficiently recover immune cells that are embedded in the LP.

In this study, we describe a new cell isolation protocol that was used to recover T- and B-lymphocytes from the LP of the oral mucosa and to analyze their phenotype and tissue residency. The oral mucosa displayed a unique composition of lymphocytes and myeloid cells, which was highly enriched in CD11b^+^Ly6C^lo^ monocytes, showed paucity of invariant NKT (*i*NKT) cells, and displayed preferential accumulation of CD4 T cells. Specifically, we identified a population of CD103^+^CD69^+^ CD4 T cells that resembled tissue-resident CD8 memory T cells in the gut (16). Notably, CD8αα T cells were non-detectable and CD103^+^CD69^+^ CD8 T cells were significantly reduced, demonstrating fundamental differences between lymphocytes in the oral and the gut mucosa. Collectively, these data provide a comprehensive picture of the immune landscape in the oral mucosa and report an effective protocol for immune cell isolation that can be used to further address immune cell function in the oral cavity.

## Materials and Methods

### Mice

C57BL/6 (B6) mice were obtained from Charles River. *Rag*-deficient (RAGKO) mice were purchased from the Jackson Laboratory. Animal experiments were performed with 8- to 14-week-old mice of both sexes. All animal experiments were approved by the NCI Animal Care and Use Committee, and all mice were cared for in accordance with NIH guidelines.

### Flow Cytometry

Data were acquired on LSR Fortessa or LSRII flow cytometers (BD Biosciences) and analyzed using FlowJo and softwares designed by the Division of Computer Research and Technology, NCI. Live cells were gated using forward scatter exclusion of dead cells stained with propidium iodide. The following antibodies were used for staining: TCRβ (H57-597), B220 (RA3-6B2), NK1.1 (PK136), CD11b (M1/70), CD44 (IM7), CD103 (2E7), and CD69 (H1.2F3), all from eBioscience; CD3 (2C11), CD4 (GK1.5), CD8α (53-6-7), TCRγδ (GL3), and CD11c (HL3), all from BD Biosciences; and CD45 (30-F11), CD8β (YTS156.7.7), and Ly6C (HK1.4) from BioLegend. CD1d tetramers loaded with PBS-57 and unloaded controls were obtained from the NIH tetramer facility (Emory University, Atlanta, GA, USA).

### Leukocyte Isolation

Liberase DL (dispase low), DH (dispase high), TL (thermolysin low), TM (thermolysin medium), and TH (thermolysin high) were purchased from Roche, and Collagenase IV from Gibco. Oral mucosal tissues were dissected from the buccogingival, sublingual, palatal areas, and tongue (Figure S1A in Supplementary Material). The average weight of extracted oral mucosa tissue per mouse was 0.201 ± 0.006 g. Tissues from oral mucosa were processed by finely chopping and enzyme digesting with Liberase (0.5 mg/ml) or collagenase IV (1.0 mg/ml) at 37°C for 40 min under continuous rotation. For leukocyte isolation by the staggered enzyme digestion method (SDTL), tissues were first treated with Liberase DL (0.5 mg/ml) for 20 min followed by Liberase TL (0.25 mg/ml) for another 20 min. Thus, total incubation time for Liberase DL was 40 min, while Liberase TL digestion was only for 20 min (Figure S2 in Supplementary Material). Protease reaction was stopped by addition of EDTA (1 mM), and digested tissues were filtered through a 70-μm cell strainer (BD Biosciences). Collected cells were passed through a density gradient with 40 and 70% Percoll (GE Healthcare) for 25 min at 2,200 rpm with no brake. Lymphocytes accumulated at the interphase, and cells were harvested, washed, and resuspended in cell culture media before further analysis. Small intestine intraepithelial leukocytes (SI IELs) were isolated as previously described (23). For isolation of lung mononuclear cells, lungs were harvested after PBS perfusion, diced into pieces, and treated identical to oral mucosa tissues but using collagenase IV (1.0 mg/ml).

### FTY720 Injection

Sex- and age-matched B6 mice were given intraperitoneal injections of 125 μg of FTY720 (Cayman Chemical), three times a week for 2 weeks. Mice were sacrificed 24 h after the last injection for analysis.

### Statistical Analysis

Data are shown as mean ± SEM. Two-tailed Mann–Whitney *U*-test was used to calculate *P*-values between the two groups. One-way ANOVA with multiple comparisons was used to compare means among more than three different groups. *P ≤ 0.05; **P ≤ 0.01; ***P ≤ 0.001 were considered statistically significant (NS, not significant).

## Results

### Optimized Protocol for Lymphocyte Isolation from the Oral Mucosa

**Figure 1:**
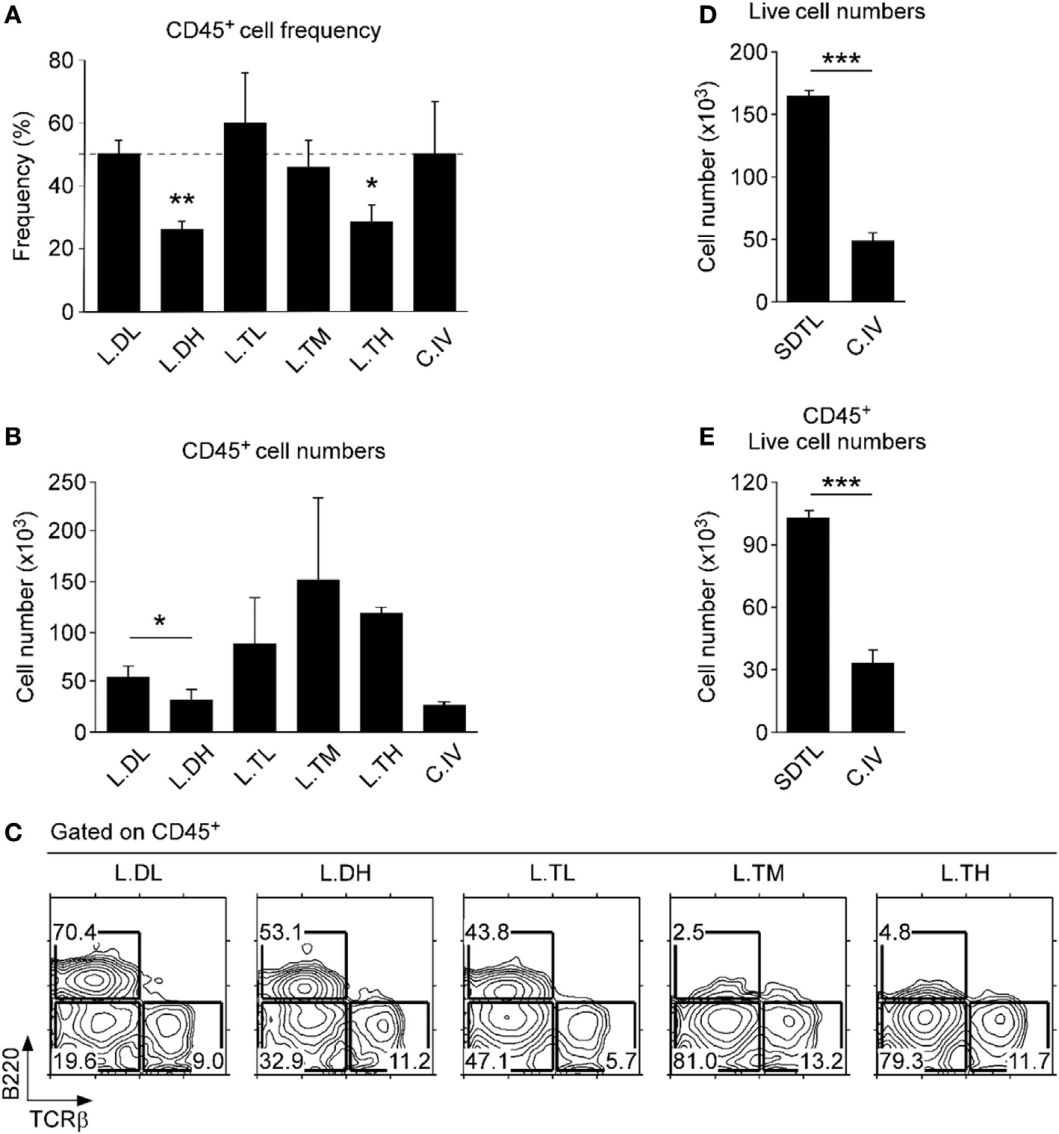
**Optimized leukocyte isolation from the lamina propria of the oral mucosa**. CD45^+^ cell frequencies **(A)** and numbers **(B)** in oral mucosa isolates that were processed using different proteolytic enzymes. Isolation efficiencies of Liberase (0.5 mg/ml) supplemented with different concentrations of either dispase (L.DL, L.DH) or thermolysin (L.TL, L.TM, L.TH) were compared to that of conventional collagenase IV (C.IV, 1.0 mg/ml) treatment. Tissues were incubated with each enzyme for 40 min. Results show mean ± SEM of 16 B6 mice in three independent experiments. **(C)** Cell surface B220 and TCRβ expression on oral mucosa isolates that were processed using different combinations of proteolytic enzymes. Results are representative of three independent experiments. Cell numbers were determined after isolation from the oral mucosa using the staggered Liberase DL and TL digestion (SDTL) or C.IV treatment. Panels **(D,E)** indicate numbers of total and CD45^+^-gated live (propidium iodide-negative) cells. Results show mean ± SEM of 11 mice with SDTL and 8 mice with C.IV digestion in three independent experiments.

Conventional methods for cell isolation from tissues are plagued by low efficiency and biased recovery against certain lymphocyte subsets (24). Thus, we first aimed to improve the recovery and efficiency of conventional isolation protocols for oral mucosa lymphocytes (25). To do so, we experimented with different concentrations and combinations of proteolytic enzymes and found a highly purified mixture of collagenase I and II, i.e., Liberase (Roche Life Science), to be highly effective in digesting oral tissues. Importantly, addition of the neutral proteases dispase and thermolysin significantly increased the efficacy of Liberase (Figures 1A,B). However, co-treatment with high concentrations of thermolysin induced a dramatic loss of surface B220 antigens and other surface proteins, such as CD4 coreceptors (Figure 1C and data not shown), so that we limited the use of thermolysin to low concentration and short incubation time. Moreover, we found that a staggered treatment of Liberase digestion, first, in addition to low concentration of dispase (L.DL) followed by addition of low concentration of thermolysin (L.TL), resulted in the most efficient recovery of CD45^+^ hematopoietic origin cells and increased numbers from the oral mucosa (Figures 1D,E; Figure S1B in Supplementary Material). This new protocol of low dosage dispase and thermolysin staggered treatment, which we refer to as SDTL method (Figure S2 in Supplementary Material), still induced degradation of some proteolysis-sensitive molecules, such as CD62L (Figure S1C in Supplementary Material) (26). Nonetheless, we found SDTL method consistently and reproducibly superior to conventional methods regarding viability and cell recovery of CD45^+^ hematopoietic cells from the oral mucosa (Figures 1D,E). Collectively, this new method permitted us to assess the lymphocyte composition of the oral mucosa in a highly effective and reproducible manner.

### Lymphocyte Subsets in the Oral Mucosa

**Figure 2:**
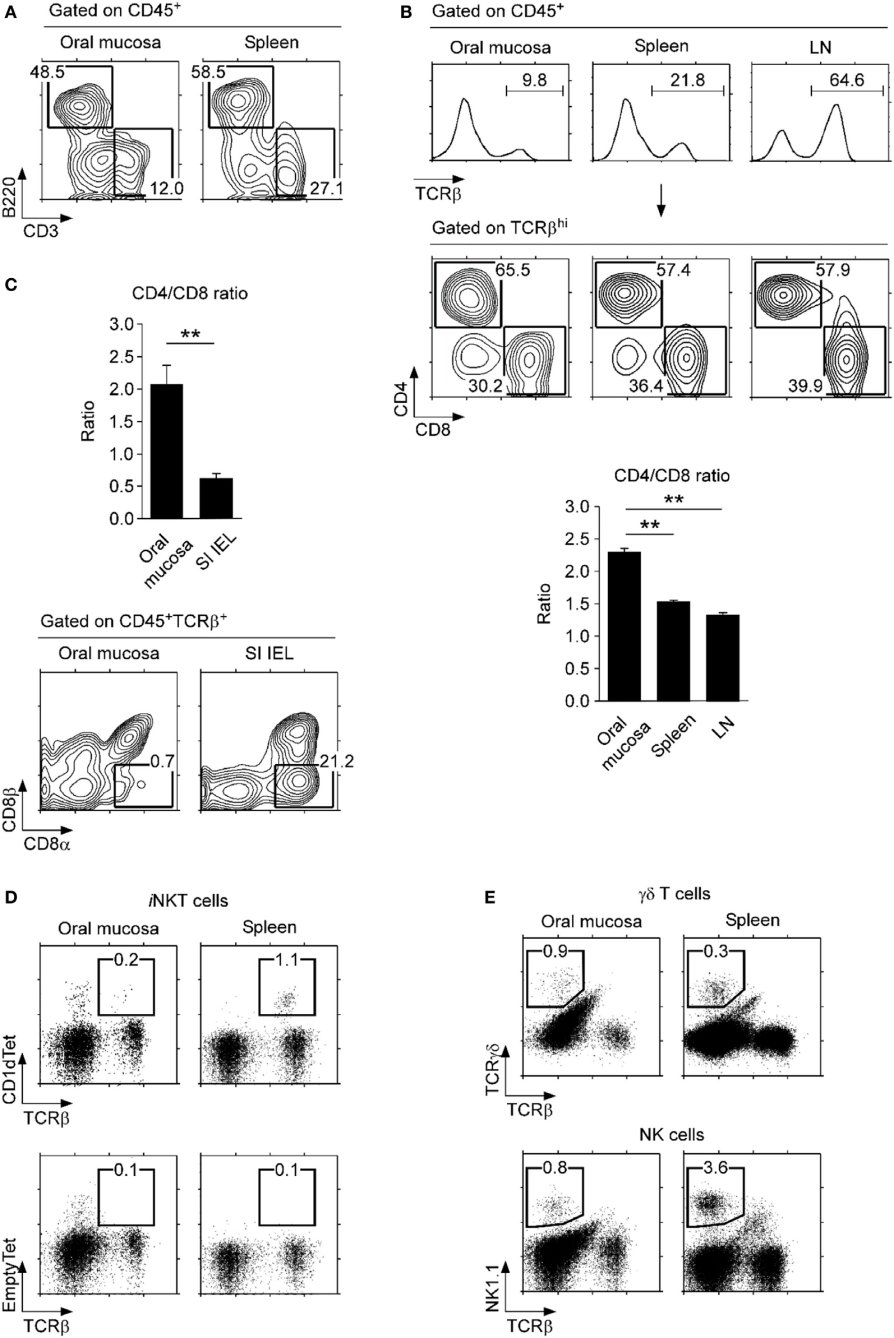
**Lymphocyte subsets in the oral mucosa**. **(A)** B220^+^ and CD3^+^ cell frequencies in CD45^+^-gated cells from the oral mucosa. Results are representative of three independent experiments. **(B)** T cell frequencies (top) and CD4/CD8 ratios (bottom) in the indicated organs. Results are representative and the bar graphs show summary of seven independent experiments. **(C)** CD4/CD8 ratio (top) and CD8α, CD8β analysis of CD45^+^TCRβ^+^ cells in the oral mucosa and small intestine intraepithelial leukocyte (SI IEL) (bottom). Results are representative of three independent experiments. **(D)** Invariant NKT (*i*NKT) cells were identified in the oral mucosa and spleen using PBS57-loaded CD1d tetramers (top) and empty-CD1d tetramers as control (bottom). Results are representative of three independent experiments. **(E)** γδ T cells (top) and NK cells (bottom) in the oral mucosa and spleen. Results are representative of three independent experiments. Oral mucosa tissues were processed under identical conditions, using the SDTL protocol outlined in Figure S2 in Supplementary Material.

Compared to lymphocytes isolated from the spleen, we found a modest decrease in B220^+^ B lineage lymphocytes and a marked decrease in CD3^+^ T lineage cells among CD45^+^ cells in the oral mucosa (Figure 2A). In fact, the oral mucosa contained a significantly lower fraction of αβ T cells compared to secondary lymphoid organs (SLOs), such as spleen and lymph node (LN) (Figure 2B). The loss was more pronounced for CD8 T cells so that the CD4/CD8 ratio in the oral mucosa was the highest among analyzed tissues (Figure 2B, bottom). These data indicated a preference for CD4 T cells in the oral mucosa which contrasted to the mucosal tissue of the small intestine, where CD8 T cells preferentially accumulate (Figure 2C, top) (16). Notably, the increase of CD8 T cells among SI IEL was due to the accumulation of CD8αα T cells and was specific to the gut mucosa (Figure 2C, bottom). Indeed, comparative analysis of the oral mucosa to the lung, which represents another protective mucosal tissue where T cells reside (Figure S3A in Supplementary Material) (27), showed that both oral and lung mucosal tissues contained lower frequencies of CD8 T cells relative to spleen and gut mucosa (Figure S3B in Supplementary Material).

Interestingly, we also found that *i*NKT cells were absent from the oral mucosa. This was quite surprising as *i*NKT cells are easily found in most, if not all tissues, including lung, liver, fat tissues, and also in all SLOs (Figure 2D) (28, 29). In comparison, other lymphocyte subsets, such as γδ T cells and NK cells, were present in significant numbers in the oral mucosa (Figure 2E), so the paucity of *i*NKT cells is selective. The biological significance of *i*NKT cell exclusion from the oral mucosa is not yet clear to us.

### Myleoid Cell Subsets in the Oral Mucosa

**Figure 3:**
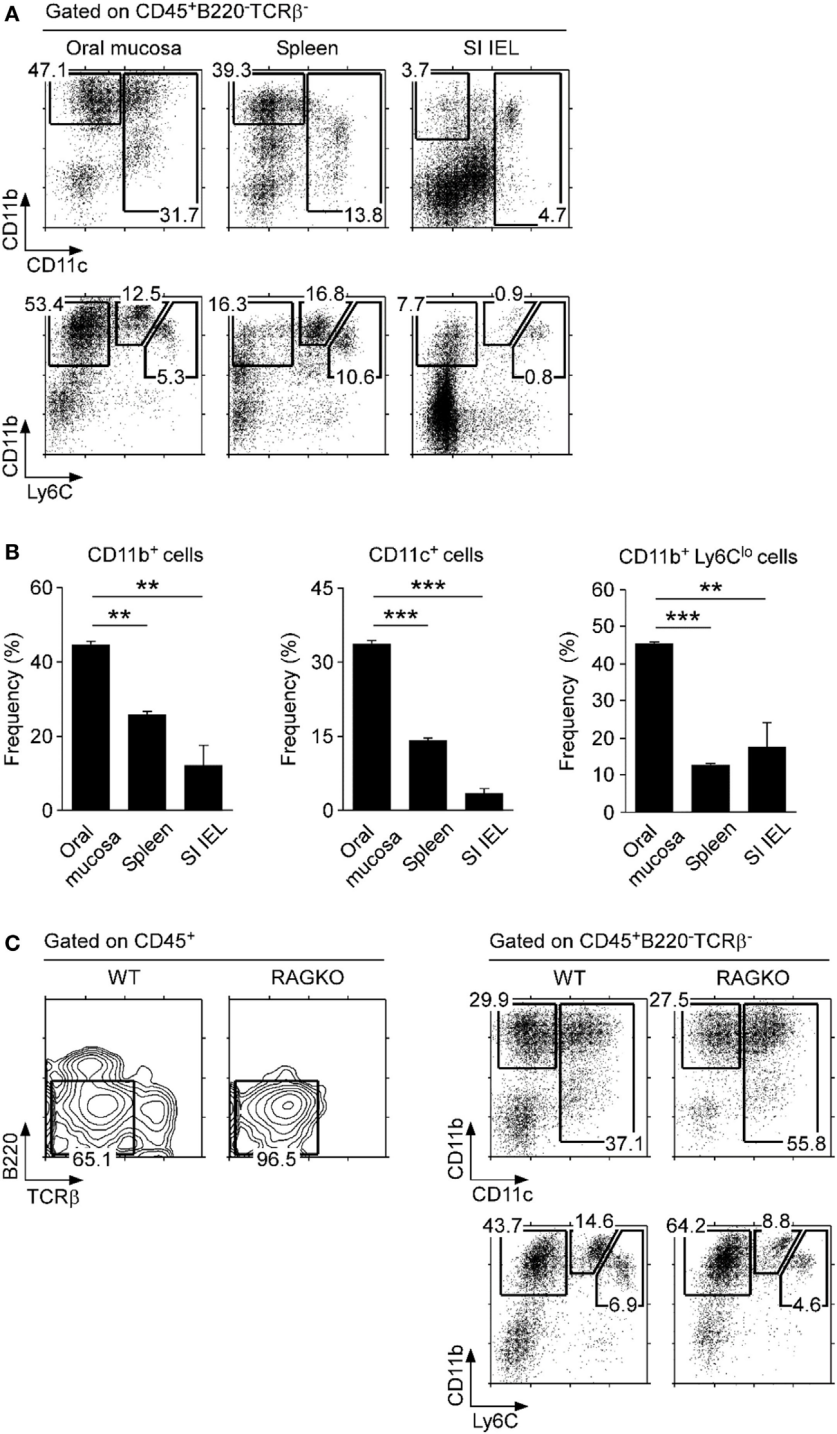
**Myeloid cell subsets in the oral mucosa**. **(A)** CD11b, CD11c, and Ly6C expression on CD45^+^-gated non-B, non-T cells in the indicated organs. Results are representative of five independent experiments. **(B)** Frequencies of CD11b^+^, CD11c^+^, and CD11b^+^Ly6C^lo^ cells in the oral mucosa. Data show summary of five independent experiments. **(C)** CD11b, CD11c, and Ly6C expression on CD45^+^-gated non-B, non-T cells in the oral mucosa of wild-type (WT) and RAGKO mice. Results are representative of three independent experiments.

Myleoid-origin monocytes, dendritic cells, and macrophages have been previously described in the oral mucosa, but frequencies of individual subsets varied dramatically among studies (9, 18, 30). In the current study, we identified myeloid cells only among CD45^+^-gated cells, which stratified the myeloid origin subsets for better identification and enumeration. We found that the non-B, non-T fraction of the oral mucosa harbored a large population of CD11b^+^ cells, which comprised macrophages and dendritic cells (Figures 3A,B) (31). Enrichment in dendritic cells was further confirmed by the concomitant increase of CD11c^+^ cells (Figure 3A, top; Figure 3B), which is an established marker for dendritic cells. Importantly, using surface expression of Ly6C, we were able to further delineate CD11b^+^ cells into three distinct subsets, CD11b^+^Ly6C^hi^, CD11b^+^Ly6C^int^, and CD11b^+^Ly6C^lo^. CD11b^+^Ly6C^hi^ cells are usually considered as inflammatory monocytes that are negative for MHC-II and Ly6G (32), and we found them being underrepresented in the oral mucosa relative to spleen and lung (Figure 3A, bottom; Figure S3C in Supplementary Material). On the other hand, we observed a marked increase in CD11b^+^Ly6C^lo^ cells, which corresponded to resident monocyte/macrophages (Figure 3B, bottom) (33). Thus, the oral mucosa is highly enriched in CD11b^+^ and/or CD11c^+^ APCs of myeloid origin, and specifically enriched in monocytes of the tissue-resident CD11b^+^Ly6C^lo^ phenotype. In this regard, the oral mucosa is similar to lung mucosa (Figure S3C in Supplementary Material). To further assess if tissue residency and recruitment of distinct monocyte subsets is dependent on lymphocytes, we analyzed the oral mucosa of *Rag*-deficient mice (RAGKO). We did not observe noticeable differences between wild-type (WT) B6 mice and RAGKO mice (Figure 3C), indicating that the monocyte population in the oral mucosa is maintained independently of the adaptive immune system.

### Tissue Residency of Oral Mucosal Lymphocytes

**Figure 4:**
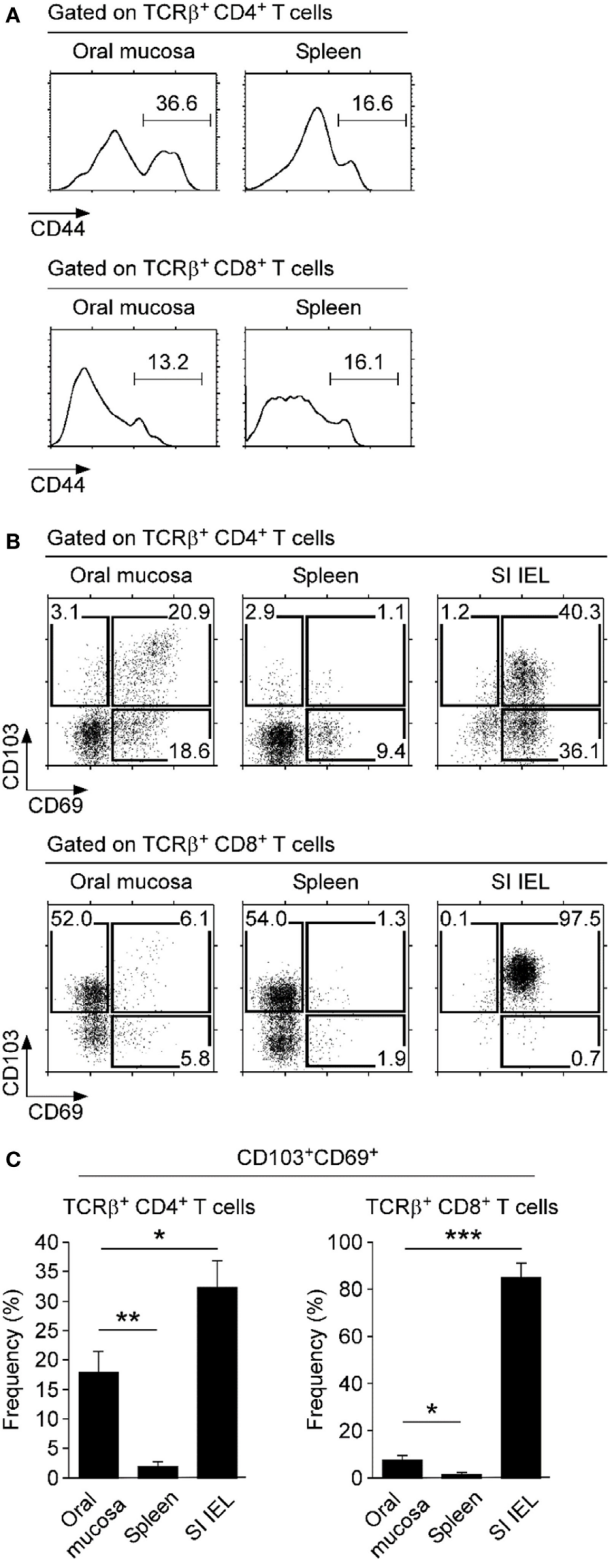
**Expression of tissue retention molecules on oral mucosa T cells**. **(A)** Surface CD44 expression on CD4 and CD8 T cells of spleen and oral mucosa. Results are representative of four independent experiments. **(B)** Surface CD69 and CD103 expression on CD4^+^ (top) and CD8^+^ T cells (bottom) isolated from the indicated organs. Results are representative of five independent experiments. **(C)** Frequency of CD103^+^CD69^+^ cells among CD4 and CD8 T cells in the indicated organs. Bar graphs show summary of five independent experiments.

To understand the immune status of oral mucosa T cells, next, we examined activation marker expression on CD4 and CD8 T cells. CD44 is a widely used activation marker that is highly induced upon TCR stimulation and memory phenotype differentiation (34). We found that most CD8 T cells in the oral mucosa were CD44^lo^ and only a small fraction was CD44^hi^ (Figure 4A, bottom). In contrast, a large fraction of oral mucosa CD4 T cells expressed high levels of CD44 (Figure 4A, top) and, interestingly, also the acute activation marker CD69 (Figure 4B, left). Moreover, many of the CD69^+^ CD4 T cells in the oral mucosa co-expressed CD103 (Figures 4B,C), an integrin with tissue retention function that binds to E-cadherin on epithelial cells (35). Co-expression of CD69 and CD103 is considered a hallmark of tissue-resident memory cells that have alarming function and serve as first responders to pathogens on site of infection (36, 37). Thus, these results showed that CD4 T cells with a tissue-resident CD103^+^ CD69^+^ phenotype were highly enriched in the oral mucosa, but interestingly not in the spleen and lung (Figure 4B, top; Figure S3D top in Supplementary Material). In contrast, CD103^+^CD69^+^ memory phenotype CD8 T cells, which accumulate in the gut mucosa (Figure 4B, bottom; Figure 4C), were conspicuously absent in the oral mucosa. In fact, CD103^+^CD69^+^ CD8 T cells were only found among SI IELs and were not represented in significant numbers neither in the spleen nor lung mucosa (Figure S3D bottom in Supplementary Material). These results suggested that either the oral mucosa lacks tissue-resident memory phenotype CD8 T cells or the tissue residency in the oral mucosa is independent of CD69 and CD103 expression.

**Figure 5:**
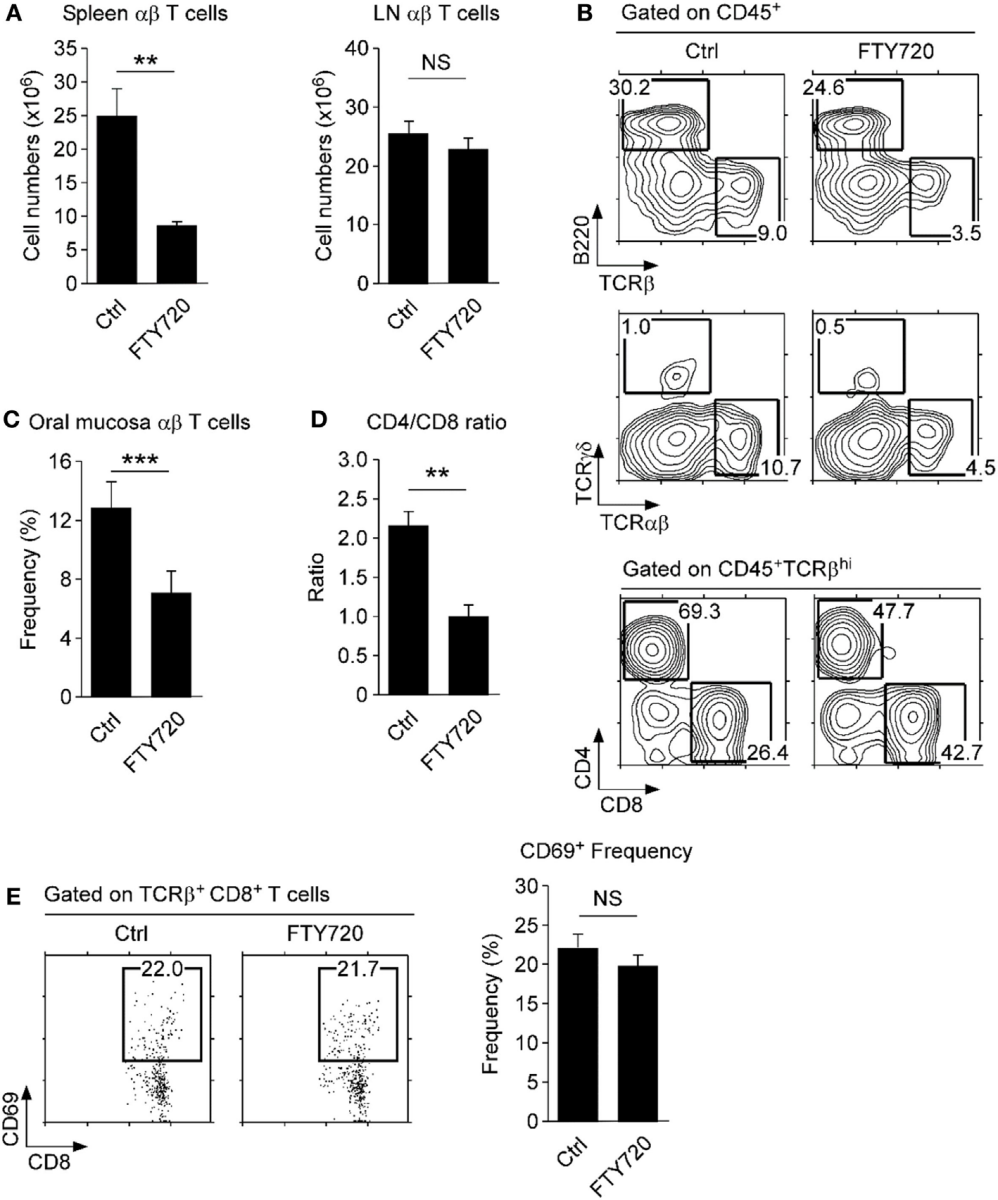
**Tissue residency of oral mucosal lymphocytes**. **(A)** αβ T cell numbers in spleen and lymph node (LN) of FTY720-treated mice. Single cell suspension of spleen and LN were assessed for T and B cell surface markers and used to determine cell numbers. Results are summary of two independent experiments with each five mice for FTY720 and vehicle-injected mice. **(B)** Phenotype of oral mucosal lymphocytes in FTY720-adminstered mice. Results are representative of two independent experiments with each five mice for FTY720 and vehicle-injected mice. **(C,D)** Frequency and CD4/CD8 ratio of αβ T cells in the oral mucosa upon FTY720 administration. Results show summary of three independent experiments with each 15 mice for FTY720 and 10 mice for vehicle control (water) injection. **(E)** CD69 surface expression on CD8 T cells upon FTY-720 or vehicle control administration. Dot plots are representative of two independent experiments. Bar graph shows frequencies of CD69^+^ cells among CD8 T cells as mean ± SEM.

Consequently, we wished to know if the oral mucosa contains tissue-resident CD8 T cells. To test this idea, we injected WT mice with the S1P1 receptor antagonist FTY720, which inhibits circulation of T cells and traps them in the LN (38). Indeed, FTY720 injection resulted in decreased T cell numbers in the spleen but retention of T cells in the LN (Figure 5A). In the oral mucosa, we found a significant decrease (~50%) in both αβ and γδ T cell frequencies upon FTY720 treatment (Figures 5B,C). Contrary to our expectation, however, we did not find increased accumulation of CD4 T cells, which we had considered tissue resident based on their CD69 and CD103 expression (Figure 5B, bottom). Instead, we found a significant increase in CD8 T cell frequency that remained tissue resident, despite being mostly absent for surface CD69 and CD103 co-expression. Consequently, FTY720 treatment markedly reduced the CD4/CD8 ratio of αβ T cells in the oral mucosa (Figure 5D). Moreover, the remaining CD8 T cells still did not show upregulation of CD69, which would have been necessary to prevent S1P1 signaling (Figure 5E) (39). These data indicated that tissue residency of CD8 T cells in the oral mucosa is controlled by mechanisms other than, or in addition to, conventional CD103 and CD69/S1P1 receptor-dependent mechanisms. Deciphering the molecular basis for these observations will contribute to our further understanding of immune surveillance and homeostasis in the oral mucosa.

## Discussion

Oral mucosal immunity is critical for host defense and local tissue protection and also plays an essential role in maintenance and surveillance of the microbiota in the oral cavity. Nonetheless, the immune cell subsets and their functions involved in these processes have remained mostly uncharacterized. A major obstacle to address these issues has been the lack of a reliable and reproducible method to isolate immune cells from the oral mucosa. Here, we established a cell isolation protocol that utilizes a staggered treatment of two different proteolytic enzymes and collagenases, followed by a 40/70% Percoll gradient. Using this method, which we refer to as the SDTL protocol, we found all major lymphocyte populations, i.e., T-, B- cells, and γδ T cells, being represented in the oral mucosa, but observed a conspicuous absence of *i*NKT cells. Whether this is a failure to recruit or to retain *i*NKT cells in these tissues needs further investigations. In addition to lymphocytes, we also detected a large fraction of myeloid origin cells among CD45^+^ leukocytes in the oral mucosa. Using the surface markers integrin CD11c and CD11b, we identified distinct dendritic cell and macrophage subsets, which showed marked differences in their distribution not only compared to SLOs in the periphery but also to the mucosal tissue in the gut and lung. We chose to compare the oral mucosa to the gut and lung mucosa because the oral cavity represents the most proximal extent of the respiratory (lung) and digestive system (gut) (40), and thus face similar antigenic and environmental challenges. The skin also represents a major protective barrier tissue, but it is structurally dissimilar to the oral mucosa as it contains adnexal structures, such as hair follicles and sweat glands, and shows distinct patterns of keratinization, so it was excluded from the current analysis (10). Altogether, our results paint a new and more complete picture of the immune cell composition in the oral mucosa than which was available before.

Of note, we need to emphasize that our current data have been acquired from the entire oral mucosa and not from distinct locations within the oral cavity. It is well documented that the composition and function of immune cells can differ depending on the anatomical area, so dendritic cells or macrophage-like APCs in the sublingual or buccal area have specialized functions and phenotypes compared to cells in other parts of the oral mucosa (21, 40, 41). As such, our study rather describes the collective immune cell composition of the oral mucosa, and we aim to address the cellular composition of specific compartments in future studies. Nonetheless, our findings were informative as it discovered the lack of CD8αα T cells in the oral mucosa, which illustrated a marked difference to the gut mucosa, and also revealed the unique presence of CD103^+^CD69^+^ tissue-resident phenotype CD4^+^ T cells, which differed from spleen and other SLOs. Collectively, the oral mucosa represents a unique site of immune cell distribution that requires further investigations to understand its role in host protection and immune pathology.

Effective recovery of immune cells from the oral mucosa is a daunting task, because cells are embedded in tissues and stay in close contact with stromal cells. Mechanical dissociation often results in cell damage and death. Enzymatic digestion of the connective tissue using collagenase, on the other hand, is a gentler method, but it can also result in degradation of surface proteins on immune cells. Collagenase is a collective term for proteases that specifically recognizes the amino acid sequence Pro–X–Gly–Pro, of which X refers to a neutral amino acid (42). The Pro–X–Gly–Pro motif is frequently found in collagen but not in other proteins, so collagenase treatment results in preferential deconstruction of the collagen-rich cell matrix with minimal effects on lymphocytes. While type IV or H collagenase has been routinely used in tissue digestion (25, 43), in our hands, collagenase IV or H were unsatisfactory for the oral mucosa because it resulted in low efficiency cell recovery. Instead, we found that a mixture of collagenase I and II, which is commercially available as a reagent called “Liberase,” was more effective in degrading the tissue matrix. Moreover, we found that the addition of small amounts of the neutral protease dispase markedly increased cell recovery, which was further improved by an additional step of the neutral protease thermolysin. We verified the efficacy and preservation of surface antigens by treating spleen and LN tissues with the same protocol and by comparing isolated lymphocytes with procedure to cells that were isolated using conventional methods. Collectively, the staggered neutral protease treatment in the presence of Liberase enabled isolation and analysis of immune cells from the oral mucosa with high efficiency and reproducibility.

Antigenic insult or pathogen invasion can trigger dramatic remodeling of the immune architecture, resulting in rapid influx and efflux of migratory cells into the oral mucosa. Interestingly, significant fractions of T cells are tissue resident and remain on site even after mobilizing signals (16, 44). The importance of tissue-resident T cells has been recently demonstrated in their role of sensing pathogens and alarming other immune cells, specifically in barrier tissues, such as the skin, lung, and the gut (36, 37). Tissue-resident T cells are marked by co-expression of the cell adhesion molecule CD103 and the activation marker CD69, which inhibits surface transport of the S1P1 receptor (45). S1P1 receptor expression is necessary for T cells to undergo chemotaxis in response to the sphingosine-derived lipid S1P, which is expressed in high concentration in the blood and thus draws T cells into circulation (44). Notably, we found a large fraction of oral mucosal CD4 T cells to co-express CD103 and CD69, suggesting them to be tissue-resident. Oral mucosal CD8 T cells, on the other hand, were mostly absent for CD103^+^CD69^+^ T cells, which differed from the gut where the vast majority of CD8αβ T cells are CD103^+^CD69^+^ tissue-resident cells (16). Interestingly, FTY720 administrations revealed that a large fraction of CD8 T cells was still tissue resident, even without co-expression of CD103 and CD69. These results suggested that, at least in the oral mucosa, CD103 and CD69 expression is not a requirement to impose tissue residency for T cells. Notably, a recent study also reported a CD69-independent retention of tissue-resident CD8 T cells in the lung, indicating that the phenotype and retention mechanisms of resident memory T cells might vary depending on the tissue environment (46).

Collectively, this study reports the immune cell composition and distribution of the oral mucosa under homeostatic conditions and thus opens up new questions on the activation and tissue residency of these cells. Utilizing the new cell isolation protocol, we expect that these inquires can be effectively answered in future studies.

## Author Contributions

J-YP and J-HP conceptualized and designed research; J-YP and HC performed experiments, analyzed data, and prepared figures and chart; J-YP, YC, and J-HP wrote the manuscript; YC and J-HP directed the study. All authors read and approved the final manuscript.

## Conflict of Interest Statement

The authors declare no potential conflicts of interest with respect to the authorship and/or publication of this article.

## References

[1] Takahashi N Oral microbiome metabolism: from “who are they?” to “what are they doing?”. J Dent Res (2015) 94(12):1628–37. 10.1177/0022034515606045 26377570

[2] Paster BJ Boches SK Galvin JL Ericson RE Lau CN Levanos VA Bacterial diversity in human subgingival plaque. J Bacteriol (2001) 183(12):3770–83. 10.1128/JB.183.12.3770-3783.2001 11371542 PMC95255

[3] Kistler JO Booth V Bradshaw DJ Wade WG Bacterial community development in experimental gingivitis. PLoS One (2013) 8(8):e71227 10.1371/journal.pone.0071227 23967169 PMC3743832

[4] Novak N Haberstok J Bieber T Allam JP The immune privilege of the oral mucosa. Trends Mol Med (2008) 14(5):191–8. 10.1016/j.molmed.2008.03.001 18396104

[5] Corthesy B Spertini F. Secretory immunoglobulin A: from mucosal protection to vaccine development. Biol Chem (1999) 380(11):1251–62. 10.1515/BC.1999.160 10614817

[6] Fabian TK Hermann P Beck A Fejerdy P Fabian G. Salivary defense proteins: their network and role in innate and acquired oral immunity. Int J Mol Sci (2012) 13(4):4295–320. 10.3390/ijms13044295 22605979 PMC3344215

[7] Gorr SU Antimicrobial peptides of the oral cavity. Periodontol 2000 (2009) 51:152–80. 10.1111/j.1600-0757.2009.00310.x 19878474

[8] Diamond G Beckloff N Ryan LK. Host defense peptides in the oral cavity and the lung: similarities and differences. J Dent Res (2008) 87(10):915–27. 10.1177/154405910808701011 18809744 PMC2730219

[9] Hovav AH. Dendritic cells of the oral mucosa. Mucosal Immunol (2014) 7(1):27–37. 10.1038/mi.2013.42 23757304

[10] Squier CA Kremer MJ. Biology of oral mucosa and esophagus. J Natl Cancer Inst Monogr (2001) 29:7–15. 10.1093/oxfordjournals.jncimonographs.a003443 11694559

[11] Dawson DV Drake DR Hill JR Brogden KA Fischer CL Wertz PW. Organization, barrier function and antimicrobial lipids of the oral mucosa. Int J Cosmet Sci (2013) 35(3):220–3. 10.1111/ics.12038 23320785 PMC3640763

[12] Mascarell L Lombardi V Zimmer A Louise A Tourdot S Van Overtvelt L Mapping of the lingual immune system reveals the presence of both regulatory and effector CD4+T cells. Clin Exp Allergy (2009) 39(12):1910–9. 10.1111/j.1365-2222.2009.03337.x 19694757

[13] Heath WR Carbone FR. The skin-resident and migratory immune system in steady state and memory: innate lymphocytes, dendritic cells and T cells. Nat Immunol (2013) 14(10):978–85. 10.1038/ni.2680 24048119

[14] Masopust D Vezys V Marzo AL Lefrancois L. Preferential localization of effector memory cells in nonlymphoid tissue. Science (2001) 291(5512):2413–7. 10.1126/science.1058867 11264538

[15] Gebhardt T Wakim LM Eidsmo L Reading PC Heath WR Carbone FR. Memory T cells in nonlymphoid tissue that provide enhanced local immunity during infection with herpes simplex virus. Nat Immunol (2009) 10(5):524–30. 10.1038/ni.1718 19305395

[16] Masopust D Choo D Vezys V Wherry EJ Duraiswamy J Akondy R Dynamic T cell migration program provides resident memory within intestinal epithelium. J Exp Med (2010) 207(3):553–64. 10.1084/jem.20090858 20156972 PMC2839151

[17] Edelson BT Kc W Juang R Kohyama M Benoit LA Klekotka PA Peripheral CD103+ dendritic cells form a unified subset developmentally related to CD8alpha+ conventional dendritic cells. J Exp Med (2010) 207(4):823–36. 10.1084/jem.20091627 20351058 PMC2856032

[18] Allam JP Duan Y Heinemann F Winter J Gotz W Deschner J IL-23-producing CD68(+) macrophage-like cells predominate within an IL-17-polarized infiltrate in chronic periodontitis lesions. J Clin Periodontol (2011) 38(10):879–86. 10.1111/j.1600-051X.2011.01752.x 21883359

[19] Capucha T Mizraji G Segev H Blecher-Gonen R Winter D Khalaileh A Distinct murine mucosal Langerhans cell subsets develop from pre-dendritic cells and monocytes. Immunity (2015) 43(2):369–81. 10.1016/j.immuni.2015.06.017 26231115

[20] Mascarell L Lombardi V Louise A Saint-Lu N Chabre H Moussu H Oral dendritic cells mediate antigen-specific tolerance by stimulating T(H)1 and regulatory CD(4+) T cells. J Allergy ClinImmunol (2008) 122(3):603–9. 10.1016/j.jaci.2008.06.034 18774396

[21] Mascarell L Saint-Lu N Moussu H Zimmer A Louise A Lone Y Oral macrophage-like cells play a key role in tolerance induction following sublingual immunotherapy of asthmatic mice. Mucosal Immunol (2011) 4(6):638–47. 10.1038/mi.2011.28 21775981

[22] Song JH Kim JI Kwon HJ Shim DH Parajuli N Cuburu N CCR7-CCL19/CCL21-regulated dendritic cells are responsible for effectiveness of sublingual vaccination. J Immunol (2009) 182(11):6851–60. 10.4049/jimmunol.0803568 19454681

[23] Goodyear AW Kumar A Dow S Ryan EP. Optimization of murine small intestine leukocyte isolation for global immune phenotype analysis. J Immunol Methods (2014) 405:97–108. 10.1016/j.jim.2014.01.014 24508527

[24] Steinert EM Schenkel JM Fraser KA Beura LK Manlove LS Igyarto BZ Quantifying memory CD8 T cells reveals regionalization of immunosurveillance. Cell (2015) 161(4):737–49. 10.1016/j.cell.2015.03.031 25957682 PMC4426972

[25] Pandiyan P Bhaskaran N Zhang Y Weinberg A. Isolation of T cells from mouse oral tissues. Biol Proced Online (2014) 16(1):4. 10.1186/1480-9222-16-4 24612879 PMC3984730

[26] Chao CC Jensen R Dailey MO. Mechanisms of L-selectin regulation by activated T cells. J Immunol (1997) 159(4):1686–94. 9257829

[27] Vareille M Kieninger E Edwards MR Regamey N. The airway epithelium: soldier in the fight against respiratory viruses. Clin Microbiol Rev (2011) 24(1):210–29. 10.1128/CMR.00014-10 21233513 PMC3021210

[28] Lee YJ Wang H Starrett GJ Phuong V Jameson SC Hogquist KA. Tissue-specific distribution of iNKT cells impacts their cytokine response. Immunity (2015) 43(3):566–78. 10.1016/j.immuni.2015.06.025 26362265 PMC4575275

[29] Lynch L Nowak M Varghese B Clark J Hogan AE Toxavidis V Adipose tissue invariant NKT cells protect against diet-induced obesity and metabolic disorder through regulatory cytokine production. Immunity (2012) 37(3):574–87. 10.1016/j.immuni.2012.06.016 22981538 PMC4991771

[30] Barrett AW Cruchley AT Williams DM Oral mucosal Langerhans’ cells. Crit Rev Oral Biol Med (1996) 7(1):36–58. 10.1177/10454411960070010301 8727106

[31] Ginhoux F Jung S. Monocytes and macrophages: developmental pathways and tissue homeostasis. Nat Rev Immunol (2014) 14(6):392–404. 10.1038/nri3671 24854589

[32] Sunderkotter C Nikolic T Dillon MJ Van Rooijen N Stehling M Drevets DA Subpopulations of mouse blood monocytes differ in maturation stage and inflammatory response. J Immunol (2004) 172(7):4410–7. 10.4049/jimmunol.172.7.4410 15034056

[33] Chorro L Geissmann F. Development and homeostasis of ’resident’ myeloid cells: the case of the Langerhans cell. Trends Immunol (2010) 31(12):438–45. 10.1016/j.it.2010.09.003 21030305

[34] Baaten BJ Li CR Deiro MF Lin MM Linton PJ Bradley LM. CD44 regulates survival and memory development in Th1 cells. Immunity (2010) 32(1):104–15. 10.1016/j.immuni.2009.10.011 20079666 PMC2858628

[35] Agace WW Higgins JM Sadasivan B Brenner MB Parker CM. T-lymphocyte-epithelial-cell interactions: integrin alpha(E)(CD103)beta(7), LEEP-CAM and chemokines. Curr Opin Cell Biol (2000) 12(5):563–8. 10.1016/S0955-0674(00)00132-0 10978890

[36] Park CO Kupper TS. The emerging role of resident memory T cells in protective immunity and inflammatory disease. Nat Med (2015) 21(7):688–97. 10.1038/nm.3883 26121195 PMC4640452

[37] Schenkel JM Fraser KA Vezys V Masopust D Sensing and alarm function of resident memory CD8(+) T cells. Nat Immunol (2013) 14(5):509–13. 10.1038/ni.2568 23542740 PMC3631432

[38] Chiba K. FTY720, a new class of immunomodulator, inhibits lymphocyte egress from secondary lymphoid tissues and thymus by agonistic activity at sphingosine 1-phosphate receptors. Pharmacol Ther (2005) 108(3):308–19. 10.1016/j.pharmthera.2005.05.002 15951022

[39] Bankovich AJ Shiow LR Cyster JG. CD69 suppresses sphingosine 1-phosophate receptor-1 (S1P1) function through interaction with membrane helix 4. J Biol Chem (2010) 285(29):22328–37. 10.1074/jbc.M110.123299 20463015 PMC2903414

[40] Cutler CW Jotwani R. Dendritic cells at the oral mucosal interface. J Dent Res (2006) 85(8):678–89. 10.1177/154405910608500801 16861283 PMC2254185

[41] Tanaka Y Nagashima H Bando K Lu L Ozaki A Morita Y Oral CD103-CD11b+ classical dendritic cells present sublingual antigen and induce Foxp3+ regulatory T cells in draining lymph nodes. Mucosal Immunol (2017) 10(1):79–90. 10.1038/mi.2016.46 27166558

[42] Birkedal-Hansen H Moore WG Bodden MK Windsor LJ Birkedal-Hansen B DeCarlo A Matrix metalloproteinases: a review. Crit Rev Oral Biol Med (1993) 4(2):197–250. 10.1177/10454411930040020401 8435466

[43] Benya RV Schmidt LN Sahi J Layden TJ Rao MC. Isolation, characterization, and attachment of rabbit distal colon epithelial cells. Gastroenterology (1991) 101(3):692–702. 10.1016/0016-5085(91)90527-R 1650317

[44] Schwab SR Cyster JG. Finding a way out: lymphocyte egress from lymphoid organs. Nat Immunol (2007) 8(12):1295–301. 10.1038/ni1545 18026082

[45] Shiow LR Rosen DB Brdickova N Xu Y An J Lanier LL CD69 acts downstream of interferon-alpha/beta to inhibit S1P1 and lymphocyte egress from lymphoid organs. Nature (2006) 440(7083):540–4. 10.1038/nature04606 16525420

[46] Takamura S Yagi H Hakata Y Motozono C McMaster SR Masumoto T Specific niches for lung-resident memory CD8+ T cells at the site of tissue regeneration enable CD69-independent maintenance. J Exp Med (2016) 213(13):3057–73. 10.1084/jem.20160938 27815325 PMC5154946

