# Supplementary figures and images for: Phenotype and Tissue Residency of Lymphocytes in the Murine Oral Mucosa
Source: Front Immunol. 2017 Mar 8;8:250. doi: 10.3389/fimmu.2017.00250 (PMC5340784; doi:10.3389/fimmu.2017.00250)

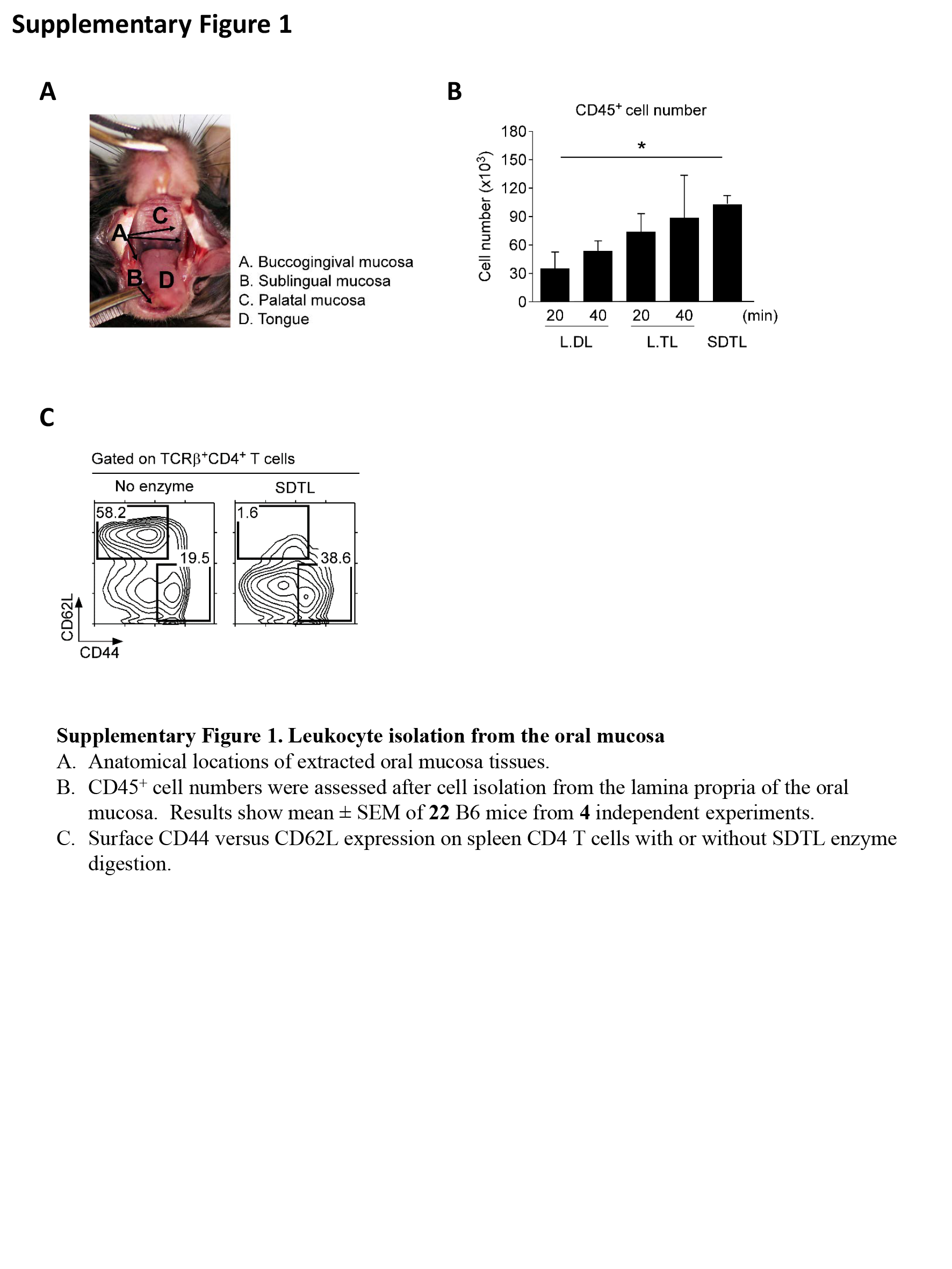

Supplement: Supplementary file 1 [file Image_1.TIF]

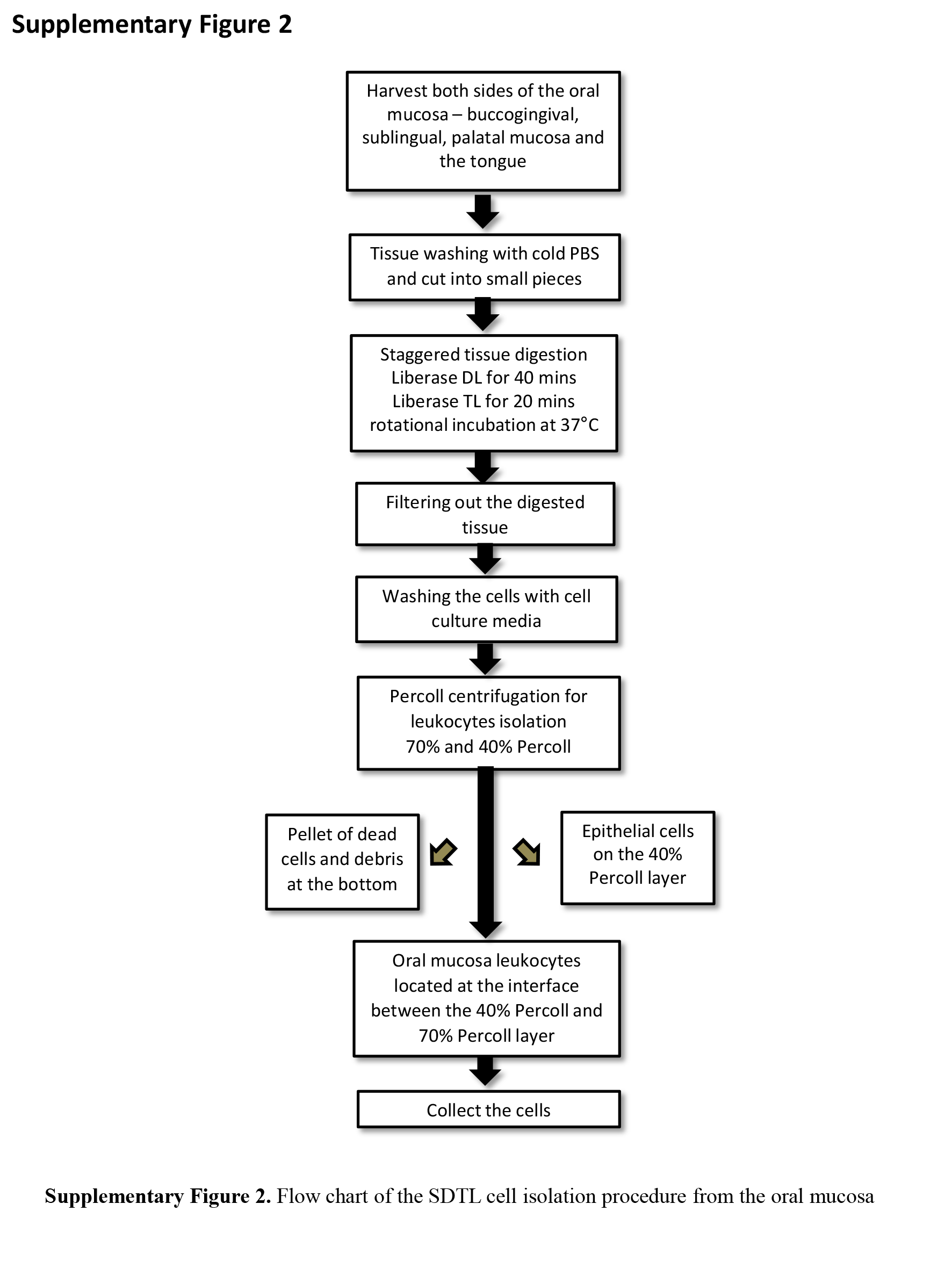

Supplement: Supplementary file 2 [file Image_2.TIF]

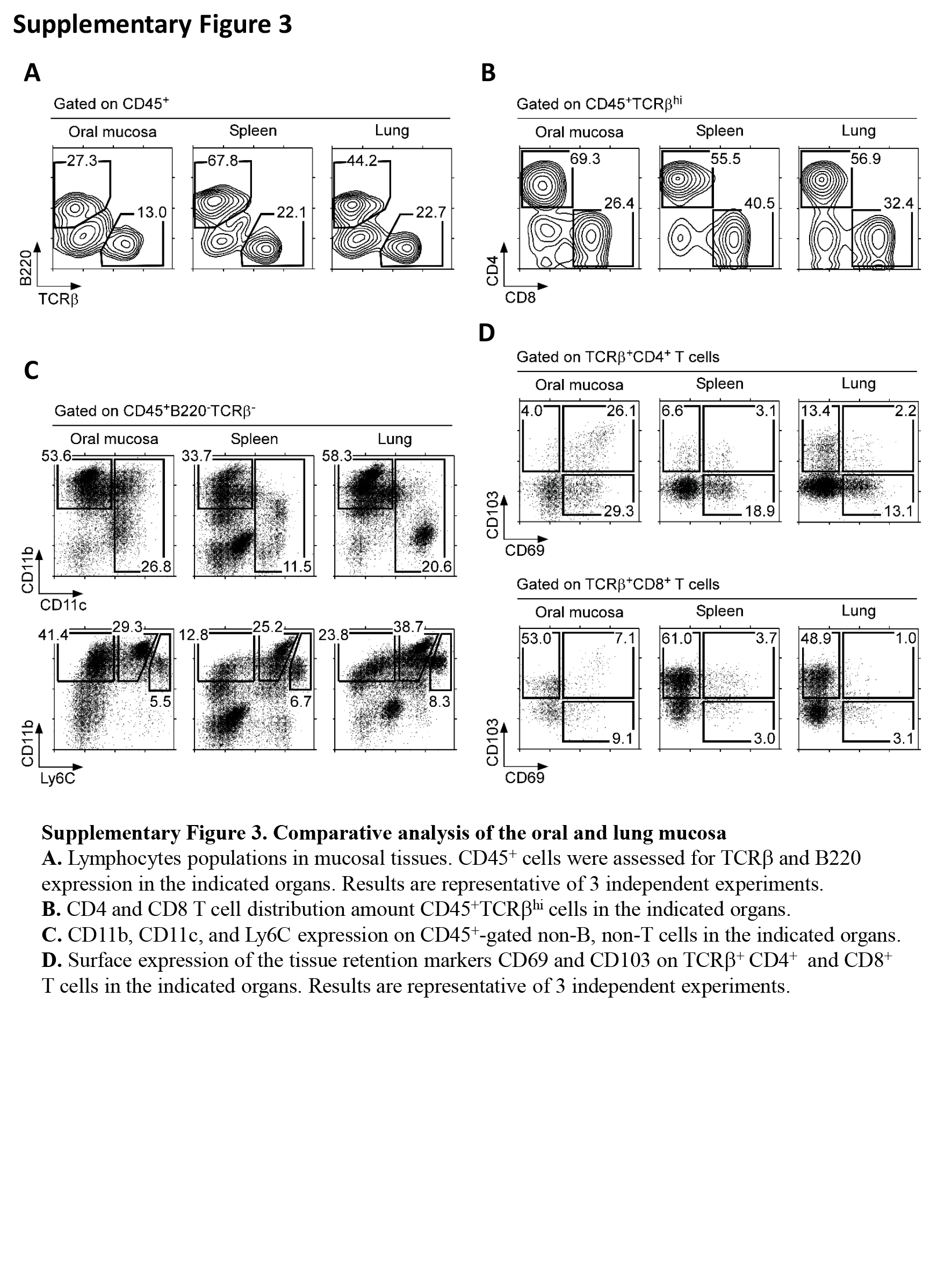

Supplement: Supplementary file 3 [file Image_3.TIF]
